# Supplementary material for: Prevalence and determinants of neonatal near miss in Ethiopia: A systematic review and meta-analysis
Source: PLoS One. 2023 Feb 21;18(2):e0278741. doi: 10.1371/journal.pone.0278741 (PMC9942950; doi:10.1371/journal.pone.0278741)
Supplement: S1 File — (DOCX) [file pone.0278741.s001.docx]

**Table 2: Newcastle - Ottawa quality assessment scale (adapted for cross sectional studies)**

| Author [year] | Selection | Comparability | Outcome | | | | | Remark |
| --- | --- | --- | --- | --- | --- | --- | --- | --- |
|  | Representativeness of the sample | Sample size | Non-respondents | Ascertainment of the exposure (risk factor) | Confounding factors are controlled | Assessment of the outcome | Statistical test |  |
| Belay HG et al [From Research square] | * | * | * | * | * | * | - | Good quality |
| Woldeyes Y et al [UP] | * | * | * | * | - | * | * | Good quality |

**Table 3: Newcastle - Ottawa quality assessment scale case-control studies**

| Author [year] | Selection | | | | Comparability | Outcome | | | Remark |
| --- | --- | --- | --- | --- | --- | --- | --- | --- | --- |
|  | Adequate case definition | Representativeness of cases | Selection of controls | Definition of controls | Comparability for factors | Ascertainment of exposure | Same method of ascertainment for cases and controls | Non-Response rate |  |
| Mersha A et al [2019] | * | * | * | * | * | * | * | * | Good quality |

**Table 4: Newcastle-Ottawa Quality Assessment Form for Cohort Studies**

| Author [year] | Selection | | | | Comparability (Maximum 2 stars) | Outcome | | | Remark |
| --- | --- | --- | --- | --- | --- | --- | --- | --- | --- |
|  | Representativeness of the exposed cohort | Selection of the non-exposed cohort | Ascertainment of exposure | Demonstration that outcome present at start | Comparability of cohorts controlled for confounders | Assessment of outcome | Was follow-up long enough for outcomes to occur | Adequacy of follow-up of cohorts |  |
| Tekelab T et al [2020] | * | ND | * | * | ** | * | * (5months) | * | Good quality |

**Keys:**

**Good quality:** 3 or 4 stars in selection domain AND 1 or 2 stars in comparability domain AND 2 or 3 stars in outcome/exposure domain

**Fair quality:** 2 stars in selection domain AND 1 or 2 stars in comparability domain AND 2 or 3 stars in outcome/exposure domain

**Poor quality:** 0 or 1 star in selection domain OR 0 stars in comparability domain OR 0 or 1 stars in outcome/exposure domain
