# Supplementary material for: Prevalence and determinants of neonatal near miss in Ethiopia: A systematic review and meta-analysis
Source: PLoS One. 2023 Feb 21;18(2):e0278741. doi: 10.1371/journal.pone.0278741 (PMC9942950; doi:10.1371/journal.pone.0278741)
Supplement: Attachment — Submitted filename: Response to Reviewers.docx [file pone.0278741.s003.docx]

**Date: October, 2022**

**To: PLOS ONE**

**Author’s Point-by-point responses**

I am so grateful for the constructive and teaching comments raised from the reviewers and I have given responses for comments and questions respectively. Besides, I have highlighted the correction in the main manuscript document with truck changes.

| **Reviewer’s comments** | **Author’s responses** |
| --- | --- |
| **Reviewer #2** | |
| The researchers accepted most of the suggestions, with a great improvement in the paper. I believe that some topics of the "discussion" could have been discussed more comprehensively. I reinforce the importance of the topic. | I would like to express my gratitude for your constructive comments. Now, I have tried to address the concern and rearranged the paragraphs under discussion. |
| **Reviewer #3** | |
| The manuscript has suffered many corrections, but English needs to be revised. Maybe there are texting problems (for example, from line 69-71 and from 126-127). On line 89, is the period correct? from 15-25 august 2020 only? On results, line 140-143, the sum does not match. All figures need to be checked, for example, is figure 5 actually figure 4? is figure 6 actually figure 5? On line 238-244, it must be emphasized that the bias of publication was a matter of concern. | - Thank you for your deep looking and constructive comments. Now, the comments are well incorporated in the edited manuscript accordingly on their respective lines. Besides, number of articles under result section was edited and written in clear expression as follow.   “A total of 101 articles and one article were found with electronic and other approach of searching respectively. Twenty eight were duplication and 74 articles were screened. From these 74 articles, 69 of them were excluded due to irrelevance, absence of full text or Abstract”.   - August 15-25, 2020 was the period for on line data searching. Thus, it was correct. |
